# Lessons for expanding virtual mentoring in academic medical institutions: a qualitative study among senior mentors

**DOI:** 10.1186/s12909-024-05852-x

**Published:** 2024-08-28

**Authors:** Elise D. Riley, Elizabeth Chur, Monica Gandhi, Jonathan D. Fuchs, John A. Sauceda, Lauren A. Sterling, Mallory O. Johnson

**Affiliations:** 1 Department of Medicine, University of California, San Francisco, 2540 23rd Street, Room 4902; UCSF mailbox 1272, 94143 San Francisco, CA USA; 2 San Francisco, CA USA; 3 Department of Public Health, San Francisco, CA USA

**Keywords:** Mentoring, Virtual mentoring, Remote mentoring, Distance mentoring

## Abstract

**Background:**

Virtual activities, hybrid work and virtual mentoring have become part of the ongoing milieu of academic medicine. As the shift to remote mentoring continues to evolve, it is now possible to adapt, refine, and improve tools to support thriving mentoring relationships that take place virtually. This study explores strategies for virtual mentoring as a cornerstone for effective training programs among senior mentors participating in an ongoing mentoring program.

**Methods:**

We conducted a qualitative study among prior and current participants of an ongoing “Mentoring the Mentors” program about key strategies for optimizing virtual mentoring. Data were coded and analyzed following a thematic analysis approach.

**Results:**

Respondents were mostly female (62%), white (58%), and associate (39%) or full professors (32%). We found that, with the expansion of hybrid and fully remote work, there are now fewer opportunities for informal but important chance meetings between mentors and mentees; however, virtual mentoring provides opportunities to compensate for reduced interactivity normally experienced in the workplace. The heightened need to plan and be more deliberate in the virtual sphere was woven throughout narratives and was the foundation of most recommendations. Specifically, a central obstacle for respondents was that spontaneous conversations were harder to initiate because virtual conversations are expected to have set agendas.

**Conclusions:**

Developing new ways to maintain meaningful interpersonal relationships in a virtual training environment, including opportunities for serendipitous and informal engagement, is critical to the success of virtual mentoring programs.

## Background

Early career guidance takes many forms that achieve different goals. For example, coaching has been associated with a shorter-term performance focus, while mentoring has a longer-term holistic focus in which the mentor has direct experience in the setting where the mentee works [1]. As an enduring relationship that seeks to help an inexperienced individual navigate their career path, mentoring is a cornerstone in training researchers and medical professionals in academic medicine, providing essential guidance, support, and valuable networking opportunities for navigating the complex research and medical practice landscape [2, 3]. Mentoring supports personal development, career guidance, and research productivity, including publication and grant funding [4].

As technology and virtual platforms have increased over the past decade, so too has virtual mentoring (i.e., any mentoring activity that does not occur face-to-face, which can include video conferencing, telephone, email, and text messaging). Virtually engaging mentees allows for mentoring across institutions and geographies; virtual platforms can reduce barriers for engaging people with disabilities, which further increases accessibility and reach [5]. Virtual mentoring provides more opportunities for mentors and mentees to make learning choices; it has been endorsed by both mentors and mentees as an effective means of expanding access to mentors in the setting of academic medicine [6].

Virtual mentoring is consistent with *connectivism learning theory*, which posits that technology plays a central role in the modern-day learning process, and that our external networks are key to continuous learning [7, 8]. Connectivism suggests that technology is changing what, how, and where we learn; it promotes learning that happens outside of an individual, such as through online networks, blogs, or virtual interactions. Virtual mentoring is a relatively new model of learning with a modest level of evaluation that could benefit from examination using a connectivism lens.

While virtual mentoring has gradually become more common, the COVID-19 pandemic forced rapid innovation in this arena. Traditional face-to-face mentoring was impossible in many cases and virtual platforms were critical in maintaining relationships through this era [9]. This abrupt pivot to a primarily online environment inspired more focus on strategies for facilitating virtual mentoring. For example, Junn et al. recommend a toolkit for fostering virtual mentorship that includes three essential components: (1) a method of easy asynchronous communication, (2) a reliable method of synchronous communication, and (3) a repository to share information [9]. In addition to toolkits and platforms, mentor-based research conducted during COVID-19 highlighted the importance of considering the mental health of mentees [10, 11]. While COVID-19 necessitated a rapid transition to virtual mentoring, hybrid work has become part of the ongoing milieu of academic medicine and virtual connection has become normalized, even as the public health emergency has ended. The COVID-19 pandemic provided a natural experiment. Building on what we learned during the pandemic, we have the opportunity to test existing theories and potentially adapt, refine, and improve tools to support thriving mentoring relationships that take place partly or entirely in the virtual sphere.

Almost two decades ago, Sambunjak and colleagues called for ongoing practical recommendations for mentoring in medicine that are evidence-based, address contextual issues, and use cross-disciplinary approaches [4]. In that spirit, we investigated how the idea that technology is changing what, how, and where we learn --which is central to the theory of connectivism --changed during the COVID-19 pandemic. Specifically, we explored strategies identified during the initial years of the COVID-19 pandemic, highlighting those that may be useful for a new era of modern academic medicine. The current paper builds on prior work exploring how COVID-19 affected mentoring among experienced HIV researchers and providers across the United States [12]. That investigation found benefits of remote mentoring (e.g., logistical ease and increased enjoyment of the mentoring process), and revealed skill gaps to address (e.g., how to work with mentees in times of distress and the prioritization of mentor well-being). This study focuses specifically on strategies for virtual mentoring.

## Methods

### Procedures and participants

Since 2012, the University of California, San Francisco (UCSF) Center for AIDS Research (CFAR) *Mentoring the Mentors* (MTM) *Program* has hosted an annual two-day intensive mentor training workshop, providing high-level skills building designed primarily for experienced mid-career and senior HIV scientists. The program conducted a long-term evaluation of prior program participants, including an ongoing collection of quantitative and qualitative data about their mentoring experiences since MTM program participation. The primary purpose of data collection was program improvement rather than research. While the project was acknowledged as research activity, it did not involve human subjects as defined by the federal regulations summarized in 45 CFR 46.102(e), and therefore did not require IRB oversight (determined by the University of California San Francisco Institutional Reivew Board; IRB Determination Reference #391553).

To date, MTM has trained approximately 300 HIV mentors from across the United States, and evaluation results have previously been described [13–16]. A recent publication [12] reported challenges expressed by MTM researchers about mentoring during COVID-19 and their recommendations for effectively mentoring during this and future crises. The current study evaluates responses to additional qualitative, open-ended queries from the same survey which were specifically focused on virtual mentoring. In addition, it augments survey information with the same questions subsequently posed to additional individuals participating in the 2022 MTM workshop held in person after the pandemic subsided, using a nominal group technique.

Survey details have been reported elsewhere [12]. Briefly, in November 2020, 226 participants of prior MTM workshops (survey group) were invited by email to complete an online survey. It included demographic questions as well as open-ended questions generated by the MTM leadership team about mentoring during COVID-19. Survey respondents received a $10 gift code for an online retailer and were entered into a raffle for additional codes. Survey questions relevant to this paper included “How does your virtual/remote mentoring compare to in-person mentoring?” and “What are the strategies for virtual mentoring you’ve found particularly helpful? Any ‘lessons learned’ you could share with others?” Survey respondents provided written answers to each open-ended question.

The same questions were posed in person to the 49 participants in the 2022 MTM program (workshop group). None had participated in the prior online evaluation survey. No reimbursement was offered. The workshop was one hour long; it was one of 14 seminars, roundtables and discussion groups conducted during a two-day MTM meeting. Two facilitators used a nominal group technique, a structured method for group brainstorming [17], to obtain responses augmenting those from survey respondents. Facilitators stated the questions which were also projected on a large screen, led brainstorming and discussion, and recorded responses, which were later examined along with prior survey data.

### Data analysis

Responses to the online survey were coded by the first and second authors using a thematic analytic approach [18]. The coding authors independently read responses to each question, separately generated themes and a coding scheme, then discussed and finalized codes. Responses provided during the in-person workshop were collected by the first and last author, coded and added to survey results, then discussed virtually by all authors in attendance. Themes were determined through consensus writing and discussion.

## Results

### Participants

Among 113 mentors who completed the 2020 online survey and 49 mentors who participated in the 2022 MTM workshop (*N* = 162 total), 62% identified as female, 19% Black/African American, 11% Asian, and 11% reported Latino/a/x ethnicity (Table 1). Primary disciplines included medicine (32%), public health (20%), social/behavioral science (20%), and basic science (20%). About one-third of participants ranked as full professors, almost 40% as associate professors, and 19% as assistant professors.

**Table 1:** Participant characteristics

	Total *n* (%) (*N* = 162)	Survey Responders *n* (%) (*n* = 113)	Workshop Attendees *n* (%) (*n* = 49)
Female Male Transgender Other	100 (61.7) 53 (32.7) 1 (0.6) 1 (0.6)	67 (61.5) 39 (35.8) 1 (0.9) 2 (1.8)	33 (70.2) 14 (29.8) 0 0
Race			
Black/African American White/Caucasian Asian Native Hawaiian/Pacific Islander American Indian/Alaskan Native Other	30 (18.5) 94 (58.0) 17 (10.5) 1 (0.6) 2 (1.2) 15 (9.3)	13 (11.5) 77 (68.1) 10 (8.9) 0 2 (1.8) 8 (7.1)	17 (34.7) 17 (34.7) 7 (14.3) 1 (2.0) 0 7 (14.3)
Latino/a/x ethnicity	17 (10.5)	9 (8.4)	8 (16.7%)
Current academic rank			
Professor Associate Professor Assistant Professor Instructor Postdoctoral Fellow	52 (32.1) 63 (38.9) 30 (18.5) 1 (0.6) 2 (1.6)	42 (38.9) 46 (42.6) 16 (14.8) 0 0	10 (20.8) 17 (35.4) 14 (29.2) 1 (2.1) 2 (4.2)
Primary Discipline of Study Medicine Public Health Social/Behavioral Science Basic Science Nursing Epidemiology Other	51 (31.5) 33 (20.4) 32 (19.8) 17 (10.5) 12 (7.4) 5 (3.1) 5 (3.1)	39 (36.5) 25 (23.4) 26 (24.3) 8 (7.5) 5 (4.7) 0 4 (3.7)	12 (25.0) 8 (16.7) 6 (12.5) 9 (18.8) 7 (14.6) 5 (10.4) 1 (2.1)

### Differences between Survey and Workshop respondents

Although there was no membership overlap between the survey and workshop groups, there was substantial consistency in their responses, with several pandemic-related exceptions. 2020 survey respondents focused heavily on mental health and emotional support during a crisis (“…*many more of the mentees are experiencing a lot of anxiety and depression that I must address*”), and their need to periodically prioritize mentee mental health over the science *(“It’s actually been focusing more on personal situations that are having a big toll on mentees’ professional lives. This is more about the pandemic than virtual mentoring…”*). Survey respondents emphasized the importance of simple acts *(“…reassure and try to keep them focused on what they can do rather than spinning out of control…Empathize and be available…”*) and self-compassion (“*Our senior leadership/mentors have continued to emphasize for us the importance of being patient*,* kind and compassionate with ourselves as well as others. The reminder for a bunch of over-achievers to allow ourselves to let some things go is helpful*.”).

By contrast, 2022 in-person workshop attendees focused on a more normalized, post-crisis culture. For example, respondents were less worried when mentees exhibited behavior that might have been concerning at the height of the pandemic, such as turning off a camera during a meeting (*“…don’t get offended…sometimes people are just burned out.”*) They focused on optimizing interpersonal connection within the virtual mentoring space. For example, to address barriers to networking in virtual and hybrid meetings, they suggested new strategies *(“…taking a few minutes at the beginning of [a meeting] for each person to have…uninterrupted time to talk about themselves and what they do*.”)

### Virtual mentoring beyond the COVID era

Besides the differences described above, themes overlapped to a large extent and responses between groups were comparable. Data were therefore combined, and analysis was structured around five domains: [1] advantages and limitations of virtual mentoring, [2] intentional virtual communities, [3] meeting structure, [4] tele-empathy, and [5] self-care for mentors.

### Advantages and limitations of virtual mentoring

Respondents named a variety of virtual mentoring pros (e.g., flexibility, time efficiency, building rapport informally by “getting a glimpse into their personal lives,” mentees benefiting from multiple mentors who hail from different institutions, increased demographic and geographic diversity of mentees) and cons (e.g., less engagement, harder to gauge someone’s emotional state). They indicated that virtual mentoring is not necessarily better or worse than in-person mentoring; rather, it is different. Several noted that some mentees thrived in virtual interactions compared with those occurring in person, particularly during group meetings (e.g., mentees who rarely asked questions in a group setting posed questions in the chat; the asynchronous nature of email also provided reflection time, enhancing their ability to contribute to group discussions). A central question for many was how to make virtual mentoring as engaging as possible for both mentors and mentees.

### Intentional virtual communities

One main obstacle was the increased difficulty of initiating informal conversations, because virtual conversations are expected to have an agenda. In the absence of “running into each other at the water cooler,” a common theme was that virtual mentoring and community-building needs to be more premeditated than traditional in-person programs. Examples of creating community and “co-locating” in a virtual space, with increased opportunities for information-sharing, included suggestions like mentees creating peer mentoring groups.

Respondents also suggested many strategies for improving virtual mentoring programs. These included creating an “onboarding” or “getting started” webinar for mentors and mentees within the same institution that provides background information, overarching goals, contacts, resources, and expectations as mentees enter a new training program. Others described using video conference working sessions and writing retreats to augment ongoing mentoring programs. Even though the crisis stage of the pandemic has passed, many respondents noted that virtual mentoring requires everyone to “show up” in more deliberate ways than in the past in order to proactively build community.

Some noted the difficulty of acknowledging or congratulating mentees in meaningful ways *(“Quality wise*,* another downside is not being able to take mentees for coffee…or to celebrate a milestone”* and not “…*being able to celebrate accomplishments in person is a loss*.”) To help compensate for these limitations, respondents suggested scheduling virtual events to mark milestones (e.g., the halfway point of the program), and celebrate achievements (e.g., a publication or grant success).

### Meeting structure

Staying regularly connected with mentees, particularly through scheduled meetings, was important (*“Don’t cancel because [a] mentee thinks they ‘don’t have much to talk about.’”*) In the case of larger meetings, respondents often suggested at least some time in smaller groups (*“Break out rooms can be quite effective if given a clear focus on what to accomplish.”*) Some respondents recommended considering meeting options beyond video conferencing (“*Zoom fatigue is real*”), and offering hybrid meeting choices such as email, texting, phone, and walking phone meetings. Others noted the importance of tailoring the best scheduling options for each mentor-mentee pair, such as choosing a fixed or changing meeting schedule, and selecting the optimal time of day for meetings, particularly those held via video conference. There was a diversity of opinions on this point: some preferred video conference meetings in the morning, before they were fatigued. Others found that video conference meetings drained their energy and left them depleted for their own work, and preferred afternoon video mentoring meetings.

Respondents also had distinct opinions about *how* to hold video conference meetings. Possibilities included using multiple modalities (i.e., not just talking, but sharing videos and web-based resources); recording video conference meetings for future review or as a resource for team members unable to attend in real time; and utilizing applications beyond screen share (e.g., *Annotate* and *Whiteboard* in Zoom), to promote active learning.

Several respondents emphasized the importance of follow-up after a mentoring meeting, both to summarize discussion items and to deepen connection between meetings. They cited the importance of lowering the bar for reaching out, approximating the serendipitous hallway and breakroom encounters which often lead to valuable conversations (“…*Patience is key and have lots of follow-up*,* quick meetings to establish timelines/deliverables*” and “*Sending follow-up emails about issues / challenges that the mentee has raised has been really positive*,* and seem to be much appreciated*.”).

### Tele-empathy

Across themes and domains, respondents expressed a strong need to convey empathy, but also recognized challenges in conveying it using virtual forms of mentoring. This was especially true when developing rapport and expressing empathy in the case of new mentoring relationships. While both survey and workshop respondents emphasized that all mentoring meetings should include a social and emotional check-in, virtual meetings need to spend even more time on compassion and empathy, a practice that can be reciprocal and extend beyond the mentoring dyad (*“…it has been fascinating to see how mentees share experiences*,* coping strategies*,* and a lot of compassion – with peers and me.”*).

### Self-care for mentors

In the tradition of the airline-gone-leadership adage, “Put your own oxygen mask on first,” [19] respondents highlighted the importance of self-care for themselves and, by extension, their mentees (“*Self-care is important and will ensure that you are able to provide the best…version of [your]self during mentoring meetings*.”) Strategies for self-care included being more flexible about meeting days and times; employing 50-minute meetings instead of 60 to allow time to stand up, walk around and go outside between virtual encounters; and on days with multiple virtual meetings, turning the camera off during “less important” ones to preserve energy.

## Discussion

Among experienced, mostly mid- and senior-level academic medical researchers reflecting on their experiences and recommendations for virtual mentoring, we found a strong emphasis on the need to be more intentional and premeditated in virtual mentoring compared with face-to-face mentoring. Overarching themes included staying connected with mentees between meetings, identifying virtual options for recognizing mentee accomplishments, following up, spending more time expressing compassion, and taking time for self-care.

Findings are consistent with connectivism learning theory, which posits that knowledge is distributed across networks where connectedness informs learning, and that that nurturing and maintaining connections are needed for continual learning [7, 8]. Results reported here strongly support the theory and provide a tangible extension. Here we found that the heightened need to plan and be more deliberate in this new virtual world was woven throughout narratives and was the foundation of most recommendations. More specifically, unscheduled hallway conversations were rare during the COVID-19 pandemic and continue to be less common as academic medicine shifts to a more virtual or hybrid format. Our findings indicate the need to compensate by creating new ways for these vital, informal connections to flourish.

Respondents reported that, beyond the reduction in chance meetings between mentor and mentee, networking opportunities beyond the mentoring dyad have been greatly reduced, with particularly negative repercussions falling on early-stage investigators. Instituting new types of icebreaker introductions during group meetings, such as the principal investigator or mentor giving each attendee several minutes of uninterrupted time to talk about themselves and their work, may help early-stage professionals network, collaborate, and succeed. This helps each speaker feel truly seen and heard, which can have profound and positive influences within the framework of virtual mentoring [20].

The increased importance of tele-empathy in virtual mentoring reported here is consistent with findings from the business world. A recent *Forbes* magazine article emphasizes the need to equip the next generation with “digital soft skills” like tele-empathy, which includes direct communication, verbal cues, active listening, and conflict resolution, all accomplished via digital communication mediums [21]. These skills are essential components of virtual mentoring; mentors need to become proficient in this new skill set, then model and teach these skills to mentees [21]. At the same time, empathy can be draining [22]. While research shows that practicing specific types of empathy can mitigate potentially negative effects on mentors [22], those studies were conducted prior to COVID-19. We need additional research to better understand how employing different types of empathy influences mentors and mentees in virtual and hybrid programs.

There are several limitations to our findings, some of which have been previously described [12]. Mentors included in this study were engaged in HIV research and self-selected to participate in an intensive in-person mentor training workshop. Survey participants also took the extra step of submitting online responses to study questions. Therefore, the current sample may not be generalizable to all mentors, but may instead represent HIV mentors who are willing to spend time and energy engaging in mentoring activities and trainings beyond mentoring itself. In addition, the survey and workshop groups were recruited differently, and only the survey group was offered an incentive, which may have led to differences in groups. The inclusion of both online survey methods and in-person group solicitation of data may have also impacted one or the other group’s input in ways that were not measured. While there were no recommendations specific to HIV, and while results apply to mentoring across a range of fields, it is possible that perspectives of HIV mentors may be different than mentors in other fields of study. In addition, the online survey format and relatively brief responses may not have completely captured subtle distinctions in underlying themes. Study strengths included longitudinal access to a diverse group of experienced mentors dedicated to improving their practice and their mentees’ experience, and willingness to share that for the benefit of others.

## Conclusion

While some forms of virtual mentoring have existed for years, the COVID-19 pandemic necessitated rapid and widespread adoption of this medium for professional development. Although the COVID-19 associated public health emergency has ended, virtual mentoring has become an ongoing part of modern academic medicine. With the expansion of hybrid and fully remote work, we have more opportunities to reach mentees who were previously geographically inaccessible; however, we also have fewer opportunities for informal but important chance meetings. Virtual mentoring conducted in an intentional fashion provides opportunities to compensate and make the mentor-mentee relationship more fulfilling. Being more thoughtful and premeditated in this new era will be key. Finding new ways to develop and maintain meaningful interpersonal relationships in a virtual environment, including opportunities for serendipitous and informal engagement, is critical to the success of future virtual mentoring dyads and programs.

## Data Availability

Due to confidentiality within a relatively small group, data sharing is limited, but available upon request.

## Abbreviations

COVID: 19–Coronavirus Disease 2019
HIV: Human immunodeficiency virus
UCSF: University of California, San Francisco
CFAR: Center for AIDS Research
MTM: Mentoring the Mentors Program
